# Stuttering candidate genes DRD2 but not SLC6A3 is associated with developmental dyslexia in Chinese population

**DOI:** 10.1186/1744-9081-10-29

**Published:** 2014-09-01

**Authors:** Huan Chen, Guoqing Wang, Jiguang Xia, Yuxi Zhou, Yong Gao, Junquan Xu, Michael SY Huen, Wai Ting Siok, Yuyang Jiang, Li Hai Tan, Yimin Sun

**Affiliations:** 1 State Key Laboratory of Proteomics, Beijing Proteome Research Center, Beijing Institute of Radiation Medicine, Beijing 102206, China; 2 National Engineering Research Center for Beijing Biochip Technology, Beijing, China; 3 CapitalBio Corporation, Beijing, China; 4 Department of Anatomy, The University of Hong Kong, Hong Kong, China; 5 State Key Laboratory of Brain and Cognitive Sciences, The University of Hong Kong, Hong Kong, China; 6 School of Humanities, The University of Hong Kong, Hong Kong, China; 7 The State Key Laboratory Breeding Base-Shenzhen Key Laboratory of Chemical Biology, The Graduate School at Shenzhen, Tsinghua University, Shenzhen, China; 8 Neuroimaging Laboratory, Department of Biomedical Engineering, School of Medicine, Shenzhen University, Shenzhen, China; 9 Guangdong Key Laboratory of Biomedical Information Detection and Ultrasound Imaging, Shenzhen 518060, China; 10 Medical Systems Biology Research Center, Department of Biomedical Engineering, Tsinghua University School of Medicine, Beijing, China

**Keywords:** Dyslexia, Dopamine D2 receptor (DRD2), Solute carrier family 6, member 3 (SLC6A3), Linkage study

## Abstract

**Background:**

Dyslexia is a polygenic developmental disorder characterized by difficulties in reading and spelling despite normal intelligence, educational backgrounds and perception. Increasing evidences indicated that dyslexia may share similar genetic mechanisms with other speech and language disorders. We proposed that stuttering candidate genes, DRD2 and SLC6A3, might be associated with dyslexia.

**Methods and results:**

The study was conducted in an unrelated Chinese cohort with 502 dyslexic cases and 522 healthy controls. In total, 23 Tag SNPs covering the two genes were selected for genotyping through Tagger program. Association analysis was performed on each SNP alone and in haplotypes. One SNP markers in DRD2 showed significant association with developmental dyslexia.

**Conclusion:**

These findings indicate that polymorphism of DRD2 gene may be a risk factor of developmental dyslexia in the Chinese population.

## Introduction

Speech and language defects can be characterized as expressive (production), as receptive (comprehension) or as mixed [1]. The emergence of genetic factors implicated across multiple speech and language disorders suggested that these disorders might share similar underlying genetic mechanisms. Dyslexia, characterized by difficulties in reading and spelling despite of normal intelligence and adequate education background, is a polygenic developmental disorder affecting about 5% to 10% school-aged children in the United States [2,3]. Though language disorders such as dyslexia are quite different concept from speech disorders, in many cases, it is difficult to discriminate a language disorder from a speech disorder in a specific individual [1]. Goulandris and his colleagues conducted a comparison study between two groups of adolescents (group one with a childhood history of language impairment, group two with developmentally dyslexic) and showed that the adolescents with dyslexia were indistinguishable from those with language impairments according to their test scores of spoken and written language skills. Moreover, both dyslexic adolescents and those with language impairments showed deficiency in phonological awareness [4]. Besides, some studies revealed that motor defects may occur in both defects and therefore might explain how the two defects are connected [5,6]. As such, genes contributing to a speech disorder are recognized as putative candidate genes for a language disorder as well.

An example is the discovery of forkhead box P2 (FOXP2) and its target genes. FOXP2 gene, which encodes a neurally expressed transcription factor, was discovered through linkage analysis of a large family who had developmental verbal dyspraxia (DVD) or childhood apraxia of speech (CAS) [7,8]. Subsequent reports provided additional evidence indicating FOXP2 genetic variants as risk factors for other speech disorders and language disorders including specific language impairment (SLI) and dyslexia, and suggested that FOXP2 acts as an important hub in networks with relevance to speech and language disorders [9-14]. Contactin associated protein-like 2 (CNTNAP2) gene, a downstream target gene of FOXP2, encodes a cell-surface neurexin protein with crucial roles in brain development. Recent studies have shown that variants of the CNTNAP2 gene are risk factors for speech and language disorders including SLI, stuttering and dyslexia [15-18]. Moreover, as reviewed in a recent study, the dyslexia candidate genes including KIAA0319, doublecortin domain containing 2 (DCDC2) and roundabout homolog 1 (ROBO1) are candidate genes involved in speech sound disorder (SSD) as well [19].

Dopamine, a monoamine neurotransmitter, is released from synaptic vesicles and regulates motivation, locomotion, cognition and reward-associated functions. Dopamine transporter (DAT), the major regulator of extracellular dopamine levels in the brain, controls the amplitude, spatial and temporal dimensions of the dopaminergic responses [20]. Dysregulation of dopaminergic system has been implicated in a variety of pathological conditions such as schizophrenia, Parkinsonism, attention deficit hyperactivity disorder and drug addiction [20-22]. Dopaminergic function is considered to be critical for modulation of the neural activity of striato-thalamo-cortical circuit, which is involved in complex goal-directed or context-dependent changes in human speech and bird song output [23]. Moreover, dopaminergic system also plays an important role in maintaining linguistic functions such as speech fluency and reading, and a number of genetic polymorphisms in the system have been identified as important risk factors [24,25]. For instance, the dopamine transporter gene (SLC6A3/DAT) has been implicated in the pathogenesis of several speech and language disorders, including dyslexia and stuttering [25,26]. Besides, a dyslexia susceptibility locus (DYX7) has been identified to link to dopamine D4 receptor (DRD4) region on chromosome 11p15.5 in participants of European ancestry [27]. In the mean time, association between DRD2 and stuttering has been found in the Chinese population through high-density genotyping [25].

Based on existing findings, dopaminergic genes DRD2 and SLC6A3 are believed to be candidate genes for dyslexia. In the present study, both genes were subjected to association and linkage analysis for dyslexia. Although the association of SLC6A3 with dyslexia has been reported in a western study [26], it would be worthwhile to validate the association of SLC6A3 with developmental dyslexia in Chinese population due to the substantial differences of linguistic and genetic backgrounds between Chinese and other western populations. Therefore, we selected tag SNPs covering above two genes and reported their association with developmental dyslexia in a large unrelated Chinese cohort.

## Materials and methods

### Subjects

Dyslexic cases and healthy controls were selected by the two-stage procedures, as previously described [28]. This study was approved by the ethical committee of Tsinghua University School of Medicine. All study subjects were informed with written consents. First, 6,900 primary school students from Shandong province of China were invited to take a Chinese reading test, which consists of questions on character-, word-, and sentence-level. Then, we selected 1794 students with reading scores above 87th percentile or below the 13th percentile in their grade for further investigation. Second, to assess the reading ability of these selected students, they were examined individually by a character reading test consisting of 300 Chinese characters. Then the Raven’s Standard Test was applied on all students to exclude participants with intelligence deficiency. In the end, 1024 participants (502 dyslexic cases and 522 controls) were chosen for subsequent analysis.

### SNP markers selection and genotyping

In total, 23 Tag SNPs covering DRD2 and SLC6A3 were selected for genotyping through Tagger program [29]. The parameters were minor allele frequency (MAF) over 5% and pairwise r^2^ threshold of 0.8. SNP Genotyping was performed using the Sequenom MassARRAY platform (Sequenom, San Diego, CA) in CapitalBio Corporation (Beijing, China). Briefly, saliva samples were collected and subjected to genomic DNA extraction using Oragene™ DNA self-collection kit (DNA Genotek Inc., Ottawa, Ontario, Canada) following the manufacturer’s instructions. DNA quality and quantity was determined by Nanodrop spectrophotometry (Nanodrop 1000 Spectrophotometer, Thermo Scientific, Wilmington, DE). Based on a locus-specific primer extension reaction, a locus-specific PCR reaction was designed using the MassARRAY Assay Design software package (v3.1). MALDI-TOF mass spectrometer and Mass ARRAY Type 4.0 software were applied for mass determination and data acquisition.

### Data analysis

Hardy-Weinberg equilibrium (HWE) tests were performed for all SNPs individually. SNPs with a HWE P-value of less than 0.00001 (in controls) were removed. PLINK software was applied for association analysis using additive, dominant, recessive and genotype models. Linkage disequilibrium (LD) and haplotype analyses (haplotypes with above 0.01 frequence) were conducted using Haploview software (Version 4.2), as previously described [30]. The Omnibus ANOVA test was performed using R software. In Omnibus ANOVA test, the independent variable is haplotype and the dependent variable is group (dyslexia or not). In the single marker and haplotype analysis, we build the logistic regression model using genotype/haplotype as variable. We also build the logistic regression model using genotype/haplotype as variable and using age and sex as covariate. Bonferonni correction was undertaken for the 23 SNPs that were adopted into the single site association analysis.

## Results

### Single marker analysis of SNPs within DRD2

In DRD2 gene, we genotyped 11 Tag SNPs and found nominal association (P < 0.05) of five SNPs with dyslexia in our cohort (Table 1). The allele C of rs1079727, the allele C of rs2002453, the allele C of rs2471851 and the allele G of rs11214607 were more frequent in patients with dyslexia than that in controls. SNP rs1079727 was significantly associated with dyslexia under recessive model (P = 0.009134, Odds Ratio, OR = 1.538) and in homozygous genotype (P = 0.008425, OR = 1.638). SNP rs2002453 was significantly associated with dyslexia in homozygous genotype (P = 0.04169, OR = 1.4909). SNP rs17115583 showed remarkable association with dyslexia under recessive model (P = 0.01465, OR = 0.7135) and in heterozygous genotype (P = 0.009318, OR = 0.6841). SNP rs11214607 also revealed remarkable association with dyslexia under dominant model (P = 0.01663, OR = 1.365) and in heterozygous genotype (P = 0.009318, OR = 0.6841).

**Table 1:** Association between SNPs in DRD2 and dyslexia using the additive, dominant, genotype, and the recessive models

	**SNP**	**Patient**	**Control**	**Crude OR (95%CI)**	**Unadjusted**	**Adjusted OR (95%CI)**	**Adjusted p-value**	**Bonferroni corrected p-value**
rs2440390	C Allele	930	975	1		1		
	T Allele	36	45	0.8436(0.5432-1.31)	0.4488	0.7823(0.4875-1.256)	0.3091	1
	CC	449	466	1		1		
	CT	32	43	0.7724(0.4800-1.2427)	0.2870905	0.7092(0.4247-1.1843)	0.18903	1
	TT	2	1	2.0757(0.1877-22.9491)	0.5513879	1.8799(0.1540-22.9427)	0.62095	1
	Dom			0.802(0.5033-1.278)	0.3533	0.7372(0.446-1.218)	0.2342	1
	Rec			2.116(0.1913-23.42)	0.541	1.928(0.1574-23.6)	0.6076	1
**rs1079727**	T Allele	521	606	1		1		
	C Allele	445	414	**1.254(1.048-1.501)**	**0.01345**	**1.337(1.1-1.626)**	**0.003592**	0.0828
	TT	143	174	1		1		
	CT	235	258	1.10831(0.8350-1.4711)	0.4765734	1.0949(0.8061-1.4871)	0.561809	1
	CC	105	78	**1.6380(1.1347-2.3645)**	**0.0084253**	**1.8827(1.2633-2.8059)**	**0.001883**	0.0437*
	Dom			1.231(0.9421-1.609)	0.1277	1.269(0.9509-1.694)	0.1056	1
	Rec			**1.538(1.113-2.127)**	**0.009134**	**1.816(1.272-2.592)**	**0.001017**	0.023*
rs2002453	T Allele	476	539	1		1		
	C Allele	490	475	1.169(0.9793-1.396)	0.0838	**1.238(1.022-1.5)**	**0.02946**	0.6785
	TT	123	136	1		1		
	CT	230	267	0.9525(0.7049-1.2869)	0.7511201	0.9340(0.6743-1.2938)	0.681255	1
	CC	130	104	1.3821(0.9692-1.9710)	0.073918	**1.5311(1.0454-2.2424)**	**0.028655**	0.6601
	Dom			1.073(0.8079-1.425)	0.6269	1.1(0.81-1.494)	0.5414	1
	Rec			**1.427(1.063-1.916)**	**0.01806**	**1.633(1.183-2.253)**	**0.002867**	0.0667
rs2471851	A Allele	565	641	1		1		
	C Allele	399	377	**1.205(1.004-1.446)**	**0.04551**	**1.269(1.041-1.549)**	**0.01864**	0.4278
	AA	165	198	1		1		
	AC	235	245	1.1510(0.8757-1.5128)	0.3131888	1.1008(0.8188-1.4799)	0.524907	1
	CC	82	66	**1.4909(1.0151-2.1897)**	**0.0416912**	**1.7458(1.1457-2.6601)**	**0.009509**	0.2185
	Dom			1.223(0.9439-1.585)	0.1276	1.225(0.9262-1.62)	0.1548	1
	Rec			1.376(0.9685-1.955)	0.07486	**1.67(1.134-2.459)**	**0.009401**	0.2162
rs12800853	C Allele	921	959	1		1		
	T Allele	43	61	0.7304(0.4877-1.094)	0.1276	0.723(0.4698-1.113)	0.1403	1
	CC	439	451	1		1		
	CT	43	57	0.7750(0.5107-1.1762)	0.2310594	0.7807(0.4999-1.2194)	0.276544	1
	TT	0	2	NA		NA		
	Dom			0.7487(0.4947-1.133)	0.1711	0.7476(0.4802-1.164)	0.1978	1
	Rec							
rs17115583	G Allele	560	549	1		1		
	A Allele	406	459	0.8636(0.7207-1.035)	0.1119	0.8382(0.6894-1.019)	0.07648	1
	GG	167	138	1		1		
	AG	226	273	**0.6841(0.5138-0.9108)**	**0.009318**	**0.6686(0.4892-0.9138)**	**0.01156**	0.2668
	AA	90	93	0.7997(0.5539-1.1545)	0.2328118	0.7555(0.5102-1.1189)	0.161701	1
	Dom			**0.7135(0.544-0.9356)**	**0.01465**	**0.6914(0.5152-0.9279)**	**0.01393**	0.3197
	Rec			1.012(0.7341-1.395)	0.9416	0.9593(0.6786-1.356)	0.8141	1
rs11214607	T Allele	589	674	1		1		
	G Allele	377	346	**1.247(1.038-1.499)**	**0.01845**	**1.324(1.084-1.616)**	**0.005965**	0.138
	TT	177	225	1		1		
	GT	235	224	**1.3336(1.0192-1.7450)**	**0.0358259**	1.3111(0.9822-1.7501)	0.066015	1
	GG	71	61	1.4796(0.9969-2.1958)	0.0517832	**1.8187(1.1654-2.8382)**	**0.00844**	0.1932
	Dom			**1.365(1.058-1.761)**	**0.01663**	**1.398(1.062-1.84)**	**0.01696**	0.391
	Rec			1.268(0.8785-1.832)	0.2045	**1.53(1.022-2.292)**	**0.03885**	0.8947
rs12574471	C Allele	831	873	1		1		
	T Allele	135	143	0.9917(0.7683-1.28)	0.9488	0.9854(0.7477-1.299)	0.9166	1
	CC	358	373	1		1		
	CT	115	127	0.9435(0.7052-1.2621)	0.6950158	0.9483(0.6924-1.2988)	0.740855	1
	TT	10	8	1.3024(0.5083-3.3372)	0.5821073	1.2050(0.4383-3.3130)	0.717837	1
	Dom			0.9647(0.7268-1.281)	0.8037	0.9643(0.7098-1.31)	0.8162	1
	Rec			1.321(0.5171-3.376)	0.5605	1.218(0.4417-3.359)	0.703	1
rs4274224	A Allele	811	833	1		1		
	G Allele	155	187	0.8478(0.6689-1.075)	0.1723	0.8359(0.6466-1.081)	0.1713	1
	AA	338	339	1		1		
	AG	135	155	0.8735(0.6631-1.1507)	0.3362564	0.8612(0.6392-1.1602)	0.325729	1
	GG	10	16	0.6268(0.2805-1.4010)	0.2550209	0.6131(0.2577-1.4590)	0.268747	1
	Dom			0.8505(0.6507-1.111)	0.2356	0.8399(0.6288-1.122)	0.2374	1
	Rec			0.6527(0.2933-1.453)	0.296	0.6246(0.2631-1.483)	0.2862	1
rs7131056	C Allele	562	583	1		1		
	A Allele	402	437	0.9565(0.8039-1.138)	0.6159	0.9669(0.8009-1.167)	0.7259	1
	CC	166	177	1		1		
	AC	230	229	1.0709(0.8095-1.4168)	0.6313223	1.0434(0.7684-1.4168)	0.78544	1
	AA	86	104	0.8817(0.6179-1.2582)	0.4877891	0.9131(0.6228-1.3387)	0.64142	1
	Dom			1.012(0.7788-1.315)	0.9298	1.005(0.7573-1.333)	0.9743	1
	Rec			0.8478(0.6172-1.165)	0.308	0.8876(0.6292-1.252)	0.4969	1
rs72999677	G Allele	679	730	1		1		
	C Allele	287	290	1.059(0.8792-1.275)	0.5477	1.099(0.8983-1.343)	0.3599	1
	GG	245	273	1		1		
	CG	189	184	1.14457(0.8769-1.4939)	0.320458	1.1395(0.8519-1.5242)	0.378904	1
	CC	49	53	1.0302(0.6735-1.5758)	0.8909049	1.1518(0.7287-1.8206)	0.545185	1
	Dom			1.119(0.8722-1.436)	0.3766	1.143(0.8731-1.496)	0.3307	1
	Rec			0.9735(0.6461-1.467)	0.8979	1.1(0.7044-1.716)	0.6762	1

*P < 0.05, Bold data: adjusted p value < 0.05.

When the results were adjusted for age and sex, only SNP rs1079727 and SNP rs17115583 remained significant under the same model (rs1079727, P_adjusted_ = 0.001017, OR = 1.816; SNP rs17115583, P_adjusted_ = 0.01393, OR = 0.6914) and genotype (rs1079727, P_adjusted_ = 0.001883, OR = 1.8827; SNP rs17115583, P_adjusted_ = 0.01156, OR = 0.6686) as before adjustment. In addition, we found rs2002453 and rs2471851 achieved significant level under recessive model (rs2002453, P_adjusted_ = 0.002867, OR = 1.633; SNP rs2471851, P_adjusted_ = 0.009401, OR = 1.67) and in homozygous genotype (rs2002453, P_adjusted_ = 0.02866, OR = 1.5311; SNP rs2471851, P_adjusted_ = 0.009509, OR = 1.7458) after adjustment. Besides, rs11214607 was significantly associated with dyslexia after adjustment for age and sex in both recessive (P_adjusted_ = 0.03885, OR = 1.53) and dominant (P_adjusted_ = 0.01696, OR = 1.398) models, the association became significant after adjustment in homozygous genotype (P_adjusted_ = 0.008440, OR = 1.8187) other than in heterozygous genotype. After the Bonferonni correction for multiple comparisons, only SNP rs1079727 significantly associated with dyslexia under recessive models (rs1079727, P_adjusted_ = 0.023, OR = 1.8160), indicating that rs1079727 is a potential SNP marker for a risk evaluation in dyslexia.

### Single marker analysis of SNPs within SLC6A3

In SLC6A3, we genotyped 12 Tag SNPs and found nominal association of one SNP with dyslexia before adjustment (Table 2). The allele A of rs11133762 was more frequent in patients with dyslexia than that in controls. SNP rs11133762 was significantly associated with dyslexia under recessive model (P = 0.04247, OR = 1.5096). After the adjustment of age and sex, SNP rs11133762 remained significant under recessive model (P = 0.04395, OR = 1.5575). However, none SNPs was significantly associated with dyslexia after the Bonferonni correction. Thus, there was no significant finding for dyslexia with any of the SNP markers.

**Table 2:** Association between SNPs in SLC6A3 and dyslexia using the additive, dominant, genotype, and the recessive models

	**SNP**	**Patient**	**Control**	**Crude OR (95%CI)**	**Unadjusted p-value**	**Adjusted OR (95%CI)**	**Adjusted p-value**	**Bonferroni corrected p-value**
rs11133762	C Allele	552	629	1		1		
	T Allele	386	363	**1.222(1.013-1.475)**	**0.03628**	**1.232(1.004-1.511)**	**0.04568**	1
	CC	158	194	1		1		
	CT	236	241	1.2024(0.9121-1.5850)	0.19106664	1.1500(0.8517-1.5527)	0.361697	1
	TT	75	61	**1.5096(1.0141-2.2474)**	**0.04246577**	**1.5575(1.0121-2.3967)**	**0.043953**	1
	Dom			1.264(0.972-1.645)	0.08041	1.235(0.9291-1.642)	0.1461	1
	Rec			1.357(0.9429-1.954)	0.1002	1.46(0.9799-2.175)	0.06285	1
rs3863145	G Allele	925	974	1		1		
	A Allele	37	44	0.8916(0.5778-1.376)	0.604	0.935(0.5856-1.493)	0.7783	1
	GG	445	468	1		1		
	AG	35	38	0.9687(0.6011-1.5610)	0.89593367	1.0151(0.6043-1.7051)	0.954917	1
	AA	1	3	0.3506(0.0364-3.3758)	0.36433775	0.4064(0.0394-4.1922)	0.449521	1
	Dom			0.9234(0.5794-1.472)	0.7376	0.9701(0.5851-1.608)	0.9063	1
	Rec			0.3514(0.03643-3.39)	0.3658	0.4136(0.0402-4.256)	0.458	1
rs40184	C Allele	734	796	1		1		
	T Allele	232	218	1.151(0.9343-1.417)	0.1867	1.127(0.9-1.411)	0.2976	1
	CC	281	314	1		1		
	CT	172	168	1.1440(0.8763-1.4937)	0.32260995	1.0956(0.8214-1.4613)	0.53434	1
	TT	30	25	1.3409(0.7701-2.3350)	0.29988039	1.3203(0.7261-2.4007)	0.362334	1
	Dom			1.17(0.9067-1.509)	0.228	1.128(0.8567-1.485)	0.3909	1
	Rec			1.277(0.7396-2.204)	0.3804	1.306(0.7249-2.355)	0.3738	1
rs6869645	C Allele	939	987	1		1		
	T Allele	27	33	0.8604(0.5137-1.441)	0.5677	0.8694(0.4988-1.515)	0.6214	1
	CC	456	478	1		1		
	CT	27	31	0.9130(0.5365-1.5539)	0.73717584	0.9431(0.5296-1.6794)	0.842233	1
	TT	0	1	NA		NA		
	Dom			0.8845(0.5216-1.5)	0.6486	0.9032(0.5099-1.6)	0.7273	1
	Rec			NA		NA		
rs40358	A Allele	617	643	1		1		
	C Allele	347	375	0.9625(0.7976-1.161)	0.6899	0.9412(0.768-1.154)	0.5595	1
	AA	190	199	1		1		
	AC	237	245	1.01329(0.7756-1.3235)	0.92355677	0.9603(0.7183-1.2838)	0.784396	1
	CC	55	65	0.8862(0.5879-1.3360)	0.56412977	0.8738(0.5617-1.3593)	0.549582	1
	Dom			0.9866(0.7644-1.273)	0.9172	0.945(0.7171-1.245)	0.6877	1
	Rec			0.8798(0.6-1.29)	0.5123	0.8838(0.5843-1.337)	0.5587	1
rs10052016	A Allele	861	923	1		1		
	G Allele	101	93	1.167(0.8654-1.575)	0.3109	1.098(0.7937-1.52)	0.5712	1
	AA	385	418	1		1		
	AG	91	87	1.1356(0.8206-1.5715)	0.44286861	1.0895(0.7663-1.5492)	0.632995	1
	GG	5	3	1.8095(0.4297-7.6209)	0.41883352	1.3168(0.2851-6.0832)	0.724443	1
	Dom			1.158(0.8417-1.594)	0.3674	1.098(0.7764-1.552)	0.5977	1
	Rec			1.768(0.4203-7.439)	0.4369	1.297(0.2802-6.001)	0.7396	1
rs403636	C Allele	645	677	1		1		
	A Allele	319	341	0.9808(0.8089-1.189)	0.8435	0.9492(0.7711-1.168)	0.6232	1
	CC	208	220	1		1		
	AC	229	237	1.0220(0.7860-1.3288)	0.87099199	1.0459(0.7870-1.3900)	0.756883	1
	AA	45	52	0.9153(0.5884-1.4238)	0.6946183	0.7948(0.4921-1.2836)	0.347614	1
	Dom			1.003(0.7798-1.29)	0.9826	0.9992(0.7614-1.311)	0.9952	1
	Rec			0.905(0.5946-1.377)	0.6413	0.7838(0.4989-1.232)	0.2907	1
rs2937639	T Allele	816	878	1		1		
	C Allele	150	142	1.136(0.8864-1.456)	0.3137	1.128(0.8626-1.476)	0.3783	1
	TT	344	379	1		1		
	CT	128	120	1.1752(0.8805-1.5684)	0.27303583	1.1803(0.8642-1.6120)	0.297244	1
	CC	11	11	1.1017(0.4717-2.5736)	0.82287421	1.0280(0.4145-2.5496)	0.952512	1
	Dom			1.169(0.8837-1.546)	0.2739	1.169(0.8636-1.582)	0.3123	1
	Rec			1.057(0.4541-2.462)	0.8974	0.9771(0.3924-2.433)	0.9603	1
rs3756450	A Allele	506	535	1		1		
	G Allele	454	485	0.989(0.8238-1.187)	0.9055	1.018(0.8355-1.24)	0.8615	1
	AA	124	133	1		1		
	AG	258	269	1.0287(0.7633-1.3864)	0.85244842	1.0910(0.7906-1.5055)	0.596073	1
	GG	98	108	0.9733(0.6743-1.4047)	0.8849171	1.0310(0.6914-1.5373)	0.881023	1
	Dom			1.013(0.7622-1.346)	0.9299	1.073(0.7889-1.458)	0.6547	1
	Rec			0.9549(0.7023-1.298)	0.7685	0.9685(0.6952-1.349)	0.8497	1
rs2550946	A Allele	823	886	1		1		
	G Allele	143	134	1.148(0.8909-1.479)	0.2859	1.141(0.8671-1.501)	0.3463	1
	AA	350	386	1		1		
	AG	123	114	1.1899(0.8877-1.5951)	0.24476629	1.2021(0.8760-1.6495)	0.254198	1
	GG	10	10	1.1029(0.4536-2.6813)	0.82898896	1.0040(0.3872-2.6035)	0.99349	1
	Dom			1.183(0.8902-1.572)	0.2468	1.186(0.8724-1.613)	0.2761	1
	Rec			1.057(0.4361-2.563)	0.9022	0.9507(0.3652-2.475)	0.9176	1
rs12652860	C Allele	595	615	1		1		
	A Allele	371	403	0.9467(0.7832-1.144)	0.5712	0.9343(0.761-1.147)	0.5166	1
	CC	173	174	1		1		
	AC	249	267	0.9380(0.7144-1.2314)	0.64476562	0.9233(0.6859-1.2428)	0.598692	1
	AA	61	68	0.9022(0.6020-1.3523)	0.6183072	0.8904(0.5778-1.3720)	0.598766	1
	Dom			0.9307(0.7169-1.208)	0.5898	0.9163(0.691-1.215)	0.544	1
	Rec			0.9374(0.6472-1.358)	0.7326	0.9203(0.6162-1.374)	0.6849	1
rs12654851	G Allele	536	549	1		1		
	T Allele	430	471	0.9285(0.771-1.118)	0.4343	0.9315(0.7625-1.138)	0.4869	1
	GG	137	136	1		1		
	GT	262	277	0.9389(0.7017-1.2564)	0.67158199	0.9964(0.7282-1.3633)	0.981816	1
	TT	84	97	0.8597(0.5900-1.2525)	0.43094129	0.8456(0.5610-1.2747)	0.423275	1
	Dom			0.9184(0.695-1.214)	0.5493	0.9584(0.7095-1.295)	0.782	1
	Rec			0.8964(0.649-1.238)	0.5066	0.8546(0.6038-1.21)	0.3754	1

Bold data: adjusted p value < 0.05.

### Haplotype analysis

**Figure 1:**
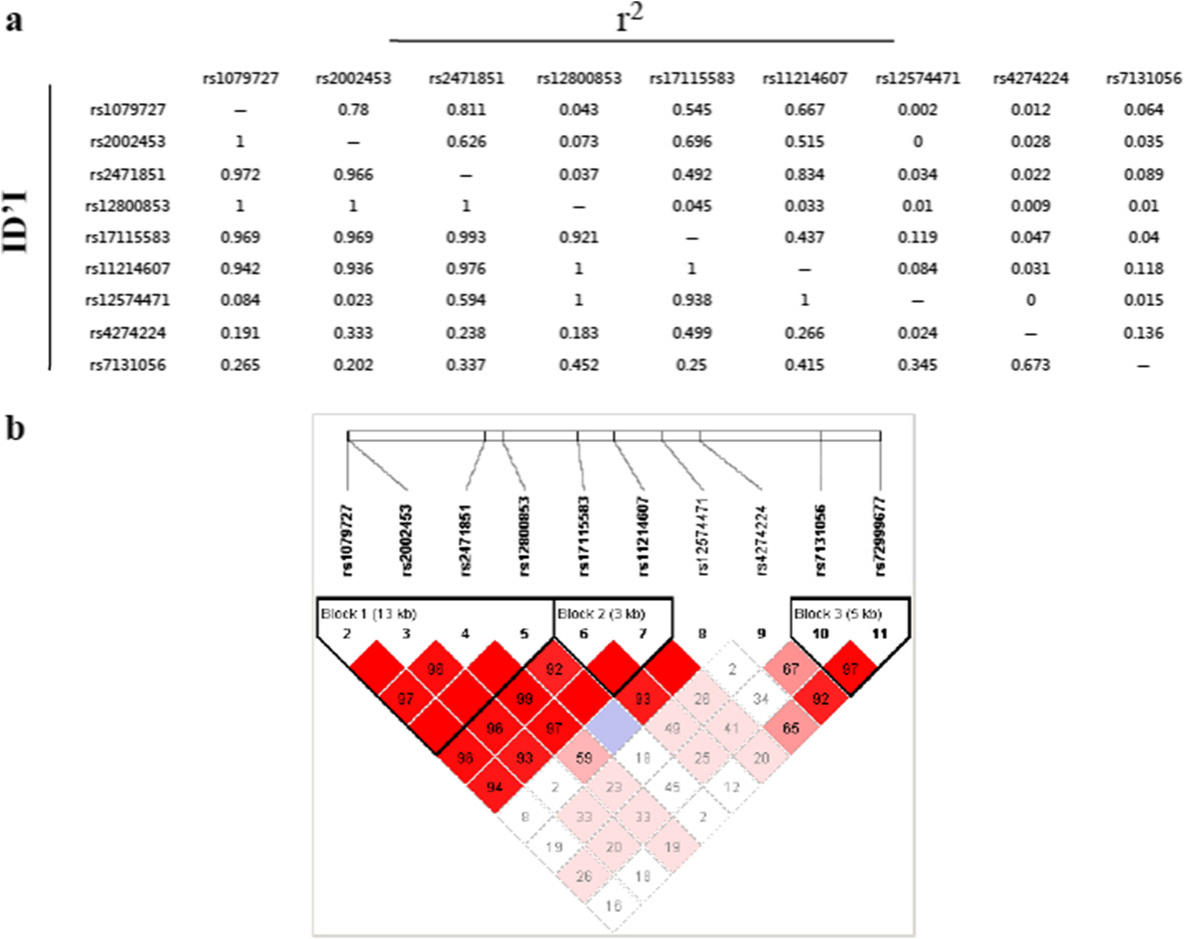
**Haplotype analysis of DRD2. (a)** Linkage disequilibrium analysis of the 11 SNPs in DRD2 investigated in healthy controls. **(b)** Three blocks were identified using Haploview software.

**Figure 2:**
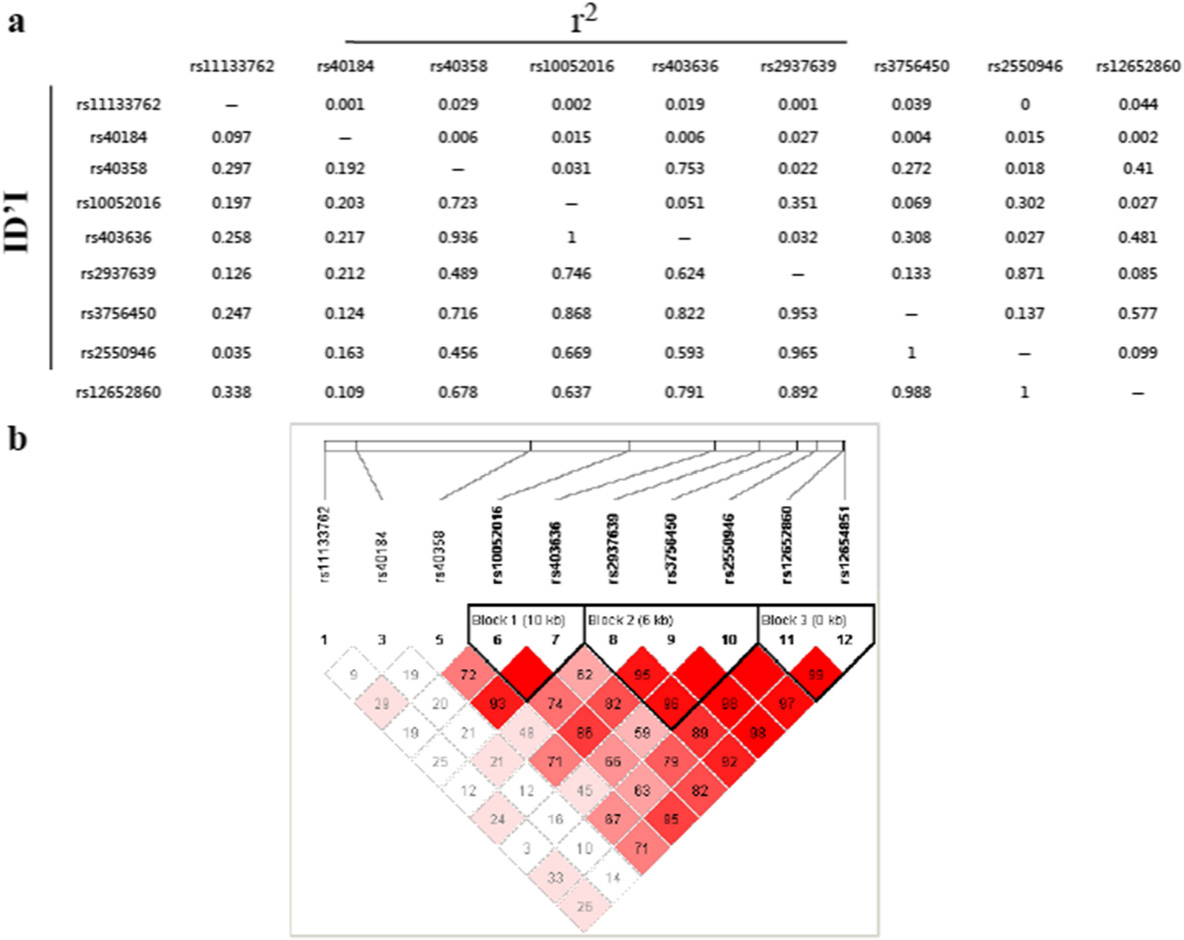
**Haplotype analysis of SLC6A3. (a)** Linkage disequilibrium analysis of the 12 SNPs in SLC6A3 investigated in healthy controls. **(b)** Three blocks were identified using Haploview software.

In DRD2, haplotype analysis was conducted in three blocks (Figure 1 and Additional file 1: Table S1). Block 1 consisting of rs1079727, rs2002453, rs2471851 and rs12800853 was associated with dyslexia (P = 0.022 Omnibus test), and included one risk haplotype CCCC (P_unadjusted_ = 0.00367, OR = 1.22). The association for haplotype CCCC remained significant after adjustment for age and sex as covariates (P_adjusted_ = 0.0146, OR = 1.28). And one protective haplotype TTAC (P_adjusted_ = 0.0327, OR = 0.812) was identified after adjustment for age and sex. Block 2 consisting of rs17115583 and rs11214607 was associated with dyslexia (P = 0.0387 Omnibus test), and included one risk haplotype GG (P_unadjusted_ = 0.0425, OR = 1.21). The association for haplotype GG also remained remarkable after adjustment for age and sex (P_adjusted_ = 0.0142, OR = 1.29). However, the above P-values failed to reach significance after the Bonferonni adjustment for multiple comparisons. Meanwhile, in SLC6A3, we identified 3 haplotypes (Figure 2), but no significant haplotype associations were found before or after adjustment (Additional file 1: Table S2).

## Discussion

Dopaminergic system has a major role in fine motor movements, and dysfunction of central dopaminergic neurotransmission has been associated with the development of speech and language [25,31-33]. Here, we aimed to examine the association between dyslexia and dopaminergic genes (dopamine receptor DRD2 and dopamine transporter SLC6A3). In DRD2 gene, using a recessive model, we demonstrated that rs1079727 was significantly associated with dyslexia with the allele C as a risk factor after Bonferonni correction. Haplotype analysis also suggested association between the risk haplotype CCCC of rs1079727-rs2002453-rs2471851-rs12800853 (Block 1) and dyslexia, but the p-value failed to reach significance after the Bonferonni correction. All four SNPs in Block1 located in the intron region of DRD2 gene, with rs1079727-rs2002453 in intron 2 and rs2471851-rs12800853 in intron 1. In previous reports, SNP rs1079727 has been associated with other common childhood-onset neurodevelopmental disorders including schizophrenia (SCZ) and attention-deficit/hyperactivity disorder (ADHD), which affected about 5% school-aged children [34-37]. Thus the significant association between SNP rs1079727 and dyslexia as well as other neurodevelopmental disorders might be an indication of its regulatory function in gene transcription regulation. To further define the loci associated with dyslexia, we also performed an imputation analysis (data not shown), which revealed that SNP rs1076560 and rs2283265 were significantly associated with dyslexia (rs1076560, P = 0.04246; rs2283265, P = 0.04196). The intronic SNP rs2283265 and SNP rs1076560 have been shown to affect alternative splicing of DRD2 transcript, and both SNPs were also associated with activity of the ventral striatum and prefrontal cortex during working memory [38]. Taken together, our data was in accordance with a recent animal study which showed that altered DRD2 expression correlated with selective cognitive impairments in working memory and behavioral flexibility [39]. Working memory represents temporary processing and storage of information, and helps to coordinate different behaviors and functions. It is well documented that dyslexic individuals showed not only impairments with language-specific skills but also working memory defects [40-42]. Hence, it is conceivable that changes in the DRD2 genotype may eventually impair the working memory of dyslexia children in our study.

SLC6A3 contains a 40 base pair variable number of tandem repeats (VNTR) in the 3'-UTR region [43]. The association between the 10-repeat SLC6A3 allele and neurodevelopmental disorders (i.e., ADHD and dyslexia) has been reported [25,43-46]. In previous studies, only several SNP markers in the 5’ region of this gene were identified to be associated with ADHD by SNP genotyping, and none were found in the 3’ region of the gene, including the 3' VNTR and the VNTR of intron 8 [47,48]. In our investigation, we only identified one SNP marker in SLC6A3 showing significant association with dyslexia after adjustment for age and sex, which located in the 3’- untranslated region (3’-UTR). However, the evidence was no longer apparent after Bonferonni adjustment for multiple comparisons. The inconsistent association of SLC6A3 with dyslexia between our study and previous western studies might be explained by linguistic and genetic differences among various populations. English is an alphabetical language while Chinese is logographic. Previous studies found that brain regions associate with dyslexia in western populations and Chinese population might be different. Dyslexia among western populations is associated with dysfunction of left temporoparietal brain regions. Differently, dyslexia among Chinese populations is associated with the left middle frontal gyrus [49,50]. Given the functional differences between these brain regions, the underlying mechanisms of dyslexia among western and Chinese populations might be different as well. But this conclusion requires further validation among larger independent Chinese dyslexia cohort.

## Conclusion

In conclusion, we found significant association between one SNP marker within DRD2 and development dyslexia in a large unrelated Chinese cohort. Our finding supports the involvement of DRD2 polymorphisms in the development of dyslexia. However, further functional analyses are required to explicate its biological roles underlying dyslexia etiology and pathology.

## Abbreviations

DRD2: Dopamine D2 receptor; SLC6A3: Solute carrier family 6, member 3 (SLC6A3); FOXP2: Forkhead box P2; DVD: Developmental verbal dyspraxia; CAS: Childhood apraxia of speech; SLI: Speech and language impairment; CNTNAP2: Contactin associated protein-like 2; DYX7: Dyslexia susceptibility locus; MAF: Minor allele frequency; HWE: Hardy-Weinberg equilibrium; LD: Linkage disequilibrium; OR: Odds Ratio.

## Competing interests

The authors declare that they have no competing interests.

## Authors’ contributions

YS and LT conceived and designed the experiments; HC, JX, GW and JX performed the experiments; YZ and YG analyzed the data; HC, GW and JX wrote the paper; MY, WS and YJ contributed reagents/materials/analysis tools; all authors read and approved the final manuscript. All authors discussed the results and commented on the manuscript.

## Supplementary Material

Additional file 1: Table S1 Haplotypes of the three blocks in DRD2 between developmental dyslexia and control subjects. **Table S2**. Haplotypes of the three blocks in SLC6A3 between developmental dyslexia and control subjects. Click here for file
