# Supplementary material for: Stuttering candidate genes DRD2 but not SLC6A3 is associated with developmental dyslexia in Chinese population
Source: Behav Brain Funct. 2014 Sep 1;10:29. doi: 10.1186/1744-9081-10-29 (PMC4236612; doi:10.1186/1744-9081-10-29)
Supplement: Additional file 1: Table S1 — Haplotypes of the three blocks in DRD2 between developmental dyslexia and control subjects. Table S2. Haplotypes of the three blocks in SLC6A3 between developmental dyslexia and control subjects. [file 1744-9081-10-29-S1.docx]

Table S1 Haplotypes of the three blocks in DRD2 between developmental dyslexia and control subjects

| Haplotype | Logistic Regression | | | | |
| --- | --- | --- | --- | --- | --- |
|  | OR | P*unadjusted* | OR | P*adjusted* | P_FDR_ |
| Block1 rs1079727-rs2002453-rs2471851-rs12800853 | | | | | |
| OMNIBUS | NA | 0.0639 | NA | **0.022** | 0.1161 |
| TCAT | 0.729 | 0.125 | 0.721 | 0.137 |  |
| **CCCC** | **1.22** | **0.0367** | **1.28** | **0.0146** |  |
| CCAC | 1.24 | 0.311 | 1.34 | 0.21 |  |
| **TTAC** | 0.856 | 0.0839 | **0.812** | **0.0327** |  |
| Block2 rs17115583-rs11214607 | | | | | |
| OMNIBUS | NA | 0.0902 | NA | **0.0387** | 0.1161 |
| **GG** | **1.21** | **0.0425** | **1.29** | **0.0142** |  |
| AT | 0.852 | 0.0837 | 0.83 | 0.0618 |  |
| GT | 0.943 | 0.613 | 0.906 | 0.433 |  |
| Block3 rs7131056-rs72999677 | | | | | |
| OMNIBUS | NA | 0.484 | NA | 0.345 | 0.5175 |
| AC | 1.03 | 0.739 | 1.07 | 0.532 |  |
| AG | 0.857 | 0.237 | 0.825 | 0.174 |  |
| CG | 1.03 | 0.756 | 1.01 | 0.903 |  |

Table S2 Haplotypes of the three blocks in SLC6A3 between developmental dyslexia and control subjects

| Haplotype | Logistic Regression | | | | |
| --- | --- | --- | --- | --- | --- |
|  | OR | P*_unadjusted_* | OR | P*_adjusted_* | P_FDR_ |
| Block1 rs10052016-rs403636 | | | | | |
| OMNIBUS | NA | 0.644 | NA | 0.825 | 0.825 |
| AA | 0.981 | 0.844 | 0.947 | 0.607 |  |
| GC | 1.15 | 0.348 | 1.08 | 0.65 |  |
| AC | 0.965 | 0.701 | 1.02 | 0.833 |  |
| Block2 rs2937639-rs3756450-rs2550946 | | | | | |
| OMNIBUS | NA | 0.717 | NA | 0.664 | 0.7968 |
| CAG | 1.19 | 0.186 | 1.17 | 0.26 |  |
| TGA | 0.996 | 0.965 | 1.02 | 0.824 |  |
| TAA | 0.949 | 0.591 | 0.922 | 0.441 |  |
| Block3 rs12652860-rs12654851 | | | | | |
| OMNIBUS | NA | 0.265 | NA | 0.285 | 0.5175 |
| CT | 0.937 | 0.493 | 0.943 | 0.568 |  |
| AG | 0.951 | 0.603 | 0.94 | 0.557 |  |
| CG | 1.23 | 0.104 | 1.24 | 0.113 |  |
